# Clinical and Clustering-Based Subtyping of Extensive Macular Atrophy With Pseudodrusen-Like Appearance (EMAP)

**DOI:** 10.1167/tvst.14.12.26

**Published:** 2025-12-24

**Authors:** Maurizio Battaglia Parodi, Alessio Antropoli, Andrea Saladino, Antonio Parciante, Sebastiano Del Fabbro, Alessandro Arrigo, Alessandro Marchese, Maria Vittoria Cicinelli, Francesco Bandello, Isabelle Audo, Lorenzo Bianco

**Affiliations:** 1 Department of Ophthalmology, IRCCS San Raffaele Scientific Institute, Milan, Italy; 2 School of Medicine, Vita-Salute San Raffaele University, Milan, Italy; 3 Eye Repair Unit, Division of Neuroscience, IRCCS San Raffaele Scientific Institute, Milan, Italy; 4 Department of Ophthalmology, Ospedale Michele e Pietro Ferrero, ASLCN2, Verduno, Italy; 5 Sorbonne Université, INSERM, CNRS, Institut de la Vision, Paris, France; 6 CHNO des Quinze-Vingts, Centre de Référence Maladies Rares REFERET and DHU Sight Restore, INSERM-DGOS CIC1423, Paris, France

**Keywords:** extensive macular atrophy with pseudodrusen-like appearance, clustering, ultra-widefield, fundus autofluorescence, macular atrophy

## Abstract

**Purpose:**

To explore the usefulness of hierarchical clustering to classify extensive macular atrophy with pseudodrusen-like appearance (EMAP) based on macular and peripheral atrophy extension.

**Methods:**

In this cross-sectional study, all participants underwent best-corrected visual acuity (BCVA) testing, optical coherence tomography (OCT), short-wavelength fundus autofluorescence (SW-AF), and ultra-widefield color fundus photography (UWF-CFP). Peripheral atrophy was quantified in degrees using OptosAdvance software, and macular atrophy area was manually measured. Based on the relative proportion of macular and peripheral atrophy, eyes were clinically classified as having predominantly central, mixed, or predominantly peripheral disease. Hierarchical clustering was performed using macular atrophy area and peripheral angular extension.

**Results:**

Eighty-five eyes from 45 EMAP patients (mean age, 64.0 ± 8.7 years; 26 females, 57.8%) were included. Peripheral pavingstone-like degeneration was observed in 57% of eyes. Four clusters were identified: Cluster 1, minimally atrophic (20%); Cluster 2, predominantly central (20%); Cluster 3, mixed (27%); and Cluster 4, predominantly peripheral (33%). Cluster 2 showed significantly worse BCVA (*P* = 0.013 vs. Cluster 1; *P* = 0.007 vs. Cluster 4) and higher prevalence of foveal atrophy (*P* = 0.004) and fibrosis (*P* < 0.0001). No significant differences in age or refractive error were found among the groups.

**Conclusions:**

Clinical and clustering-based classifications converged on consistent EMAP subtypes defined by macular and peripheral atrophy distribution. These findings suggest that combining clinical expertise with data-driven methods can help describe disease variability and guide future research.

**Translational Relevance:**

Integrating clinical expertise with unsupervised clustering may improve the identification of EMAP subtypes, aiding patient selection and outcome stratification in future trials and longitudinal studies.

## Introduction

First described in 2009 by Hamel et al.,^1^ extensive macular atrophy with pseudodrusen-like appearance (EMAP) is now recognized as a disease spectrum characterized by macular atrophy, diffuse Bruch's membrane (BM)–retinal pigment epithelium (RPE) separation, pseudodrusen-like lesions, and peripheral degeneration.^2^ It primarily affects adults of working age, with symptoms such as night blindness, photophobia, and central scotomas typically arising between the fifth and seventh decades of life, although a later onset is possible.^3^ EMAP has a poor visual prognosis, often leading to a visual acuity of 20/200 or worse within approximately 4 years from the diagnosis.^3^ Despite invariably sharing the development of macular atrophy through distinct mechanisms and with specific multimodal imaging features,^4^^–^^7^ the degree of central and peripheral retinal involvement varies across patients, from mild RPE alterations to widespread chorioretinal atrophy.^8^^,^^9^ Battaglia Parodi et al.^8^ documented that peripheral atrophy in EMAP can progress both circumferentially and centripetally, and they proposed the existence of “predominantly central” or “predominantly peripheral” subtypes. A structured classification may provide insights into disease subtypes and trajectory, allowing for a better understanding of its natural history, facilitating patient stratification in future clinical trials, and anticipating visual prognosis. However, this anatomical heterogeneity makes it particularly challenging to establish a reliable classification system for EMAP. Unsupervised machine-learning techniques provide an objective means of addressing this heterogeneity by identifying phenotypic subgroups through a data-driven framework. In this study, we applied hierarchical clustering based on macular and peripheral atrophy extension in EMAP patients, with the aim of supporting the identification of potential disease subtypes that may guide future research and patient stratification.

## Methods

### Study Design and Patient Selection

This study was a cross-sectional, observational investigation. The study adhered to the tenets of the Declaration of Helsinki. Ethical approval was obtained from the local Ethics Committee, and all patients provided signed informed consent. Data were collected from the earliest eligible visit for patients with EMAP between January 2016 and September 2024. The diagnosis of EMAP was based on the following criteria: (1) diffuse pseudodrusen and pseudodrusen-like deposits in the posterior pole and mid-periphery; (2) multilobular macular atrophy with moderately decreased autofluorescence (MDAF) signal and/or diffuse BM–RPE separation on optical coherence tomography (OCT); and (3) genetic testing negative for pathogenic variants in genes associated with inherited retinal diseases.^2^ All patients underwent a complete ophthalmological examination of best-corrected visual acuity (BCVA) on standard Early Treatment Diabetic Retinopathy Study charts, OCT and short-wavelength fundus autofluorescence (SW-AF) with a SPECTRALIS device (Heidelberg Engineering, Heidelberg, Germany), and ultra-widefield color fundus photography (UWF-CFP; Optos, Dunfermline, UK).

Eye-specific exclusion criteria were the absence of both macular atrophy and peripheral pavingstone-like degeneration (i.e., pre-atrophic disease) on UWF-CFP taken in the primary gaze position, presence of other concomitant retinal diseases (e.g., central retinal vein occlusion, diabetic retinopathy), or poor image quality due to media opacities or obstruction from the patient's eyelids or eyelashes in the photograph.

On UWF-CFP acquired in primary gaze, the extent of peripheral atrophy was measured in degrees (°) using the angular tool provided by the OptosAdvance software. Centripetal extension was assessed by measuring the distance (mm) from the optic disc to the most posterior boundary of the peripheral pavingstone-like degeneration using the caliper function of the OptosAdvance software. Peripheral atrophy patches were considered measurable only if their size met a minimum threshold of one disc diameter. If no peripheral atrophy was detected, a default value of 25 mm was assigned, corresponding to the approximate distance from the optic disc to the peripheral edge of the UWF-CFP image. The macular atrophy area, foveal thickness (FT), and subfoveal choroidal thickness (SCT) were manually measured by a single grader (AS) on SW-AF and OCT scans using the built-in Heidelberg software tools as previously described.^3^ The presence of complete RPE and outer retinal atrophy in the foveal region, macular fibrosis, and macular neovascularization (MNV) were assessed by reviewing fundus photographs and multimodal retinal imaging. Macular fibrosis was defined as a well-demarcated mound of yellowish tissue corresponding to a subretinal, compact, hyperreflective lesion on OCT.^5^

Building on observations from prior studies,^8^ two expert ophthalmologists (AA and MBP) reviewed UWF-CFP and fundus autofluorescence (FAF) images for each patient and assigned each eye to one of the following categories based on the relative proportion of macular and peripheral atrophy: (1) *predominantly central* pattern, characterized by macular atrophy dominating the clinical picture, irrespective of foveal involvement, with few or no patches of peripheral atrophy; (2) *predominantly peripheral* pattern, defined by peripheral RPE atrophy with extensive circumferential and posterior expansion with relatively limited macular involvement; and (3) *mixed* pattern, applied when neither posterior pole nor peripheral involvement clearly dominated the clinical presentation. The two graders were blinded to each other's assessments, and discordant cases were resolved through open discussion.

### Statistical Analyses

To support the proposed existence of different EMAP subtypes, hierarchical clustering was performed to identify disease subgroups and assess their consistency with the clinically determined subgroups. The area of macular atrophy and peripheral atrophy angular extension were used as core variables for clustering. Quantitative data were aggregated at the patient level by averaging eye-level measurements to account for bilateral inclusion and avoid violating the assumption of independent observations, considering the high symmetry of EMAP.^8^ Skewed variables, such as peripheral atrophy angular extension and distance from the optic disc, were log transformed. All continuous variables were standardized to their *Z*-scores before analysis.

To evaluate the appropriateness of the selected variables, additional candidates, including FT, SCT, age, and centripetal extension of peripheral atrophy, were individually tested for inclusion. Each was assessed through histogram inspection and by calculating silhouette scores in three-variable clustering models. None of these variables improved silhouette performance; therefore, they were excluded from the final model.

Hierarchical clustering was performed using Ward's method, which minimizes within-cluster variance while maximizing between-cluster variance. A dendrogram was generated to visually represent the hierarchical structure and assist in determining the optimal number of clusters. Silhouette scores were calculated to quantitatively assess cluster cohesion and separation (see Supplementary Materials).

*K*-means clustering, informed by the elbow method, was applied using the same core variables to assess the robustness and reproducibility of the identified subgroups, and χ^2^ tests were used to assess the association between generated clusters and clinically defined subtypes. Analysis of variance (ANOVA) and Kruskal–Wallis tests were performed to identify significant differences in continuous variables across clusters, and χ^2^ or Fisher's exact tests were used for categorical variables. Pairwise comparisons with the Wilcoxon rank-sum test and the Benjamini–Hochberg correction were performed when the overall ANOVA or Kruskal–Wallis test yielded *P* < 0.05. BCVA comparisons were conducted using a linear mixed model, with clusters as fixed factors and eye-level data nested within patients to account for inter-eye correlations. Continuous variables are summarized as mean ± standard deviation (SD) or median with interquartile range (IQR), and categorical variables are reported as frequencies and percentages. Statistical significance was defined as α < 0.05. All analyses were conducted using R 2023.03.0+386 (R Foundation for Statistical Computing, Vienna, Austria).

## Results

### Study Cohort

Out of 110 eyes (55 patients) with EMAP considered for the study, 25 eyes from 15 patients were excluded for central retinal vein occlusion (one eye), insufficient image quality (two eyes), absence of UWF-CFP (18 eyes), or absence of macular atrophic or peripheral pavingstone-like degeneration detected on CFP (four eyes). The final cohort was comprised of 85 eyes from 45 patients (26 females, 58%) with a mean age of 64.0 ± 8.7 years. Past medical records were available for 41 of the 45 patients, with five patients (12.2%) having a history of glaucoma and 19 patients (46.3%) diagnosed with arterial hypertension. The median BCVA (logMAR) was 0.2 (IQR, 0.1–0.7), approximately corresponding to 20/32 Snellen.

The median macular atrophy area was 13.3 µm^2^ (IQR, 5.6–20.7). On UWF-CFP captured in primary gaze, 57 of 85 eyes (67%) exhibited peripheral pavingstone-like degeneration, with a mean angular extension of 79.4° ± 48.2° and a mean distance from the optic disc of 22.1 ± 2.0 mm. Foveal atrophy was observed in 22 eyes (25.9%), and MNV complicated five eyes (5.9%), including two bilateral cases. The demographic and imaging characteristics of the cohort are summarized in Table 1.

**Table 1.** Patient Demographics and Imaging Characteristics

Characteristic	Value
Study subjects (eyes), *n*	45 (85)
Females, *n* (%)	26 (58)
Age (y), mean ± SD	64.0 ± 8.7
Arterial hypertension,^a^ *n* (%)	19 (46)
History of glaucoma,^a^ *n* (%)	5 (12)
BCVA (logMAR), median (IQR)	0.2 (0.1 to 0.7)
Refractive error (D),^b^ median (IQR)	−1.25 (−4.00 to 0.00)
Pseudophakia, *n* (%)	20 (23.5)
Macular atrophy (mm^2^), median (IQR)	13.3 (5.6 to 20.7)
Foveal atrophy, *n* (%)	22 (26)
Macular fibrosis, *n* (%)	17 (20)
MNV development, *n* (%)	5 (6)
Peripheral pavingstone-like degeneration, *n* (%)	57 (67)
Angular extension (°), median (IQR)	78 (38 to 106)
Distance from optic disc (mm), median (IQR)	16.3 (14 to 18.1)
FT (µm), mean ± SD	216.6 ± 64.2
SCT (µm), mean ± SD	131.5 ± 51.3

Eye-wise data are reported, when applicable.

a Data were obtained for 41 patients.

b Data were obtained for 57 phakic eyes.

When dividing the cohort into subgroups, only two cases required resolution through open discussion, and only one patient received a different classification between the two eyes (one classified as mixed and one as predominantly peripheral). After consultation with an additional grader (AS), the latter patient was assigned to the predominantly peripheral group in the aggregated dataset. As a result, 19 of the 45 patients (42.2%) were classified as having predominantly central involvement, 12 patients (26.7%) as mixed, and 14 patients (31.1%) as predominantly peripheral.

### Cluster Analysis

**Figure 1.**
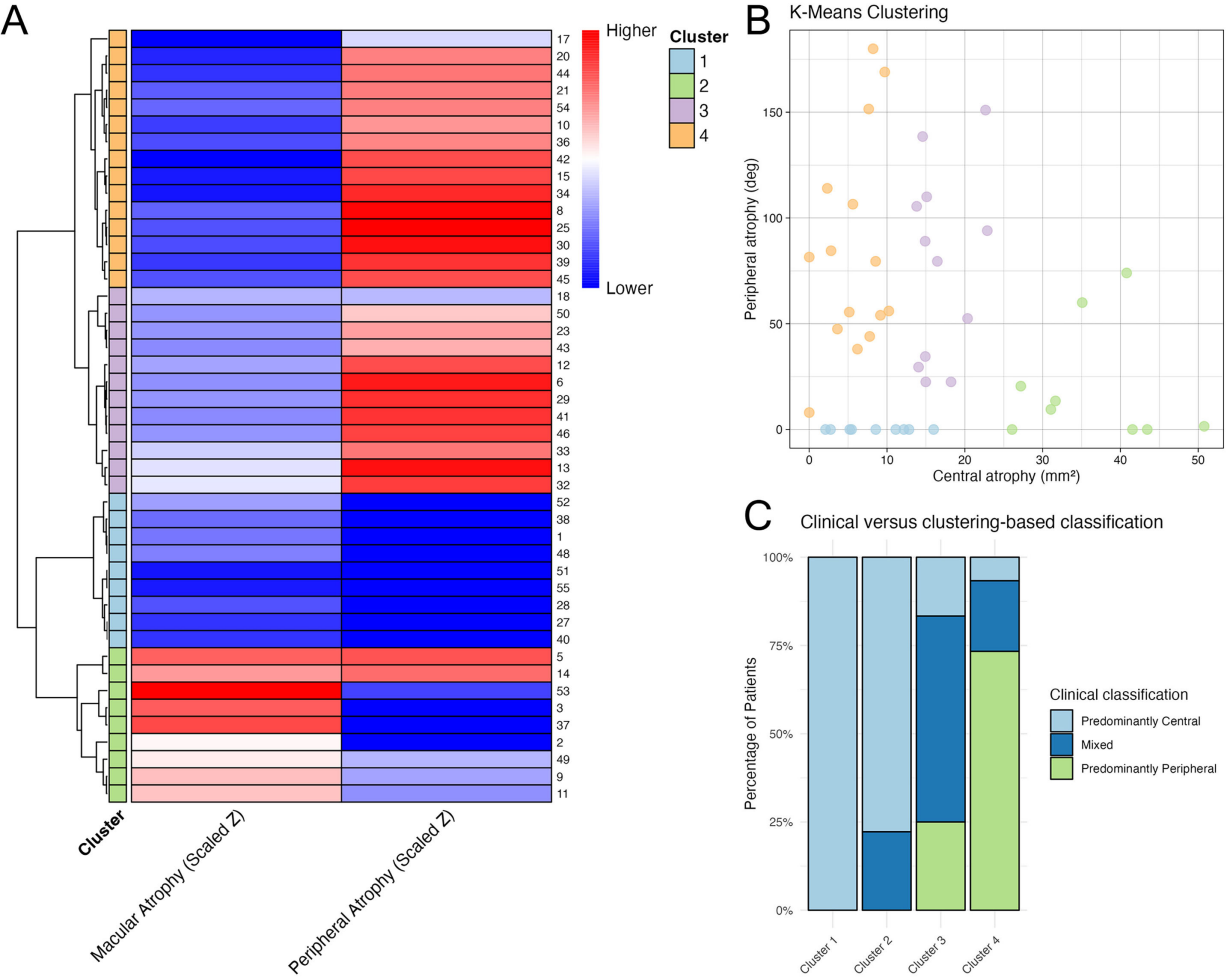
**Results of clustering analyses and concordance with the clinical classification.** (**A**) Hierarchical clustering based on macular atrophy area and angular extension of peripheral pavingstone-like degeneration. Patient IDs are shown on the right side of each row. *Z*-scores (i.e., the number of standard deviations an observation lies above or below the mean) were scaled and are displayed as a gradient from *blue* (lower) to *red* (higher). Four clusters were identified: Cluster 1, small macular atrophy with no peripheral atrophy; Cluster 2, large macular atrophy with minimal peripheral atrophy; Cluster 3, intermediate macular atrophy with extensive peripheral involvement; and Cluster 4, minimal central atrophy with extensive peripheral pavingstone-like degeneration. (**B**) Scatterplot of macular atrophy area (mm^2^, *x*-axis) versus peripheral atrophy extension (degrees, *y*-axis), color-coded by *K*-means cluster. This additional analysis corroborated the stability of the four-cluster solution, reproducing the same subgroups with identical subject distribution. Notably, data points appeared randomly distributed, showing no correlation between the two variables. (**C**) Stacked bar plots showing agreement between clinical and clustering-based classifications. The predominantly central group was split into Clusters 1 and 2, the predominantly peripheral group largely overlapped with Cluster 4, and most mixed cases were assigned to Cluster 3.

Before we proceeded with the cluster analysis, the data were aggregated at the patient level by using means for continuous variables and classifying patients as positive for binomial variables if at least one eye showed the condition. Hierarchical clustering was then conducted using the standardized values of macular atrophy area and pavingstone-like degeneration angular extension as the core variables, and four main clusters were recognized (Figs. 1A, 1B). Cluster 1 included nine out of 45 subjects (20%) and was characterized by relatively small macular atrophy and no visible peripheral atrophy. Cluster 2 was comprised of nine subjects (20%) with the largest median macular atrophy area of 35.1 mm^2^ (IQR, 31.1–41.6) and minimal or no pavingstone-like degeneration in the far periphery of the retina. Cluster 3 included 12 subjects (27%) with intermediate macular atrophy and peripheral atrophy with notable extension both circumferentially, with a median angular extension of 84.2° (IQR, 33.2°–107°), and toward the posterior pole, with a median distance from the optic disc of 17.8 mm (IQR, 14.8–19.7). Finally, Cluster 4 included 15 subjects (33%) with peripheral atrophy extension similar to Cluster 3 but with limited macular atrophy areas resembling those in Cluster 1.

**Figure 2.**
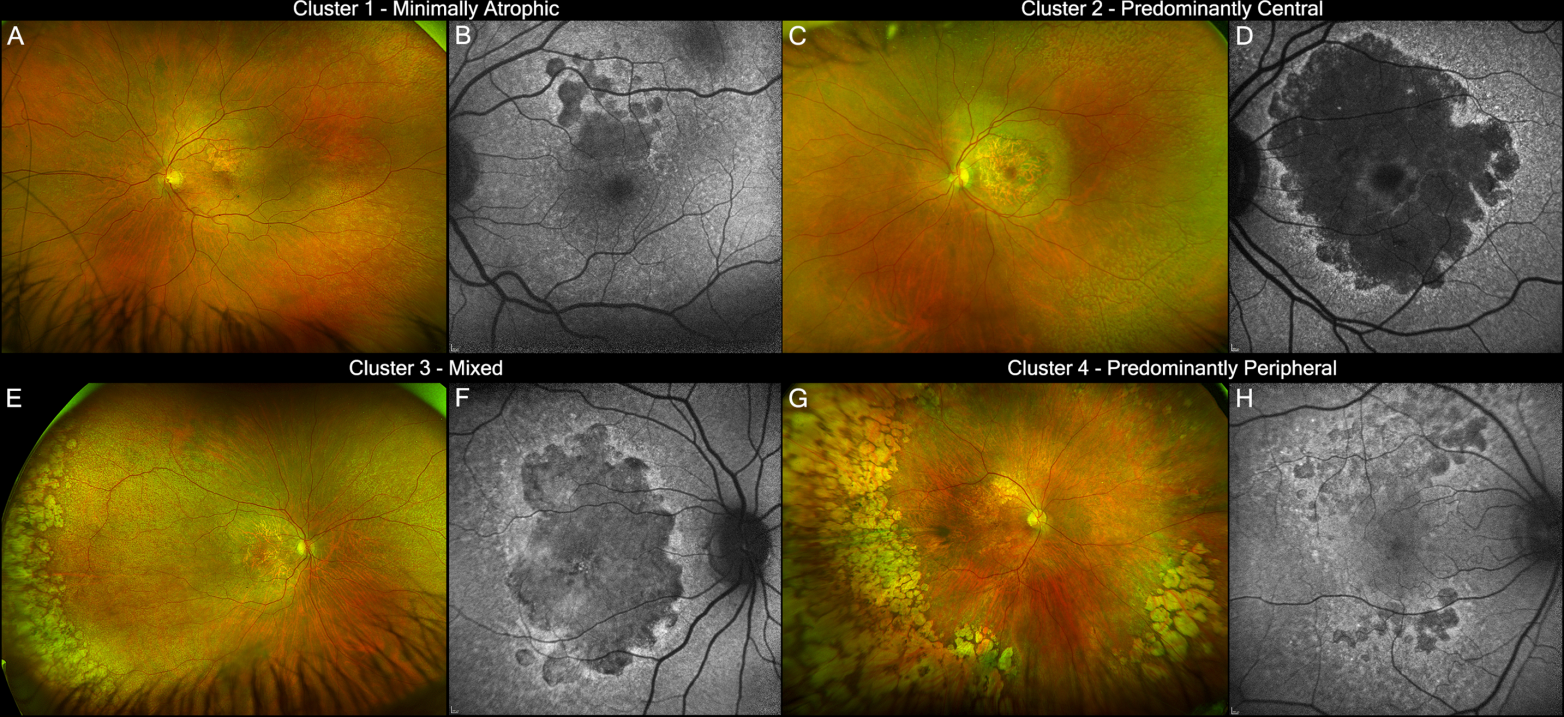
**Representative cases from each cluster.** (**A**–**H**) UWF-CFP and SW-AF images of EMAP patients from minimally atrophic (**A**, **B**), predominantly central (**C**, **D**), mixed (**E**, **F**), and predominantly peripheral (**G**, **H**) clusters.

A statistically significant association was found between the clinical and clustering-based classifications (χ^2^ = 37.7; *P* = 1.27e^–^^6^). Specifically, all patients in Cluster 1 and most patients in Cluster 2 were classified as having predominantly central disease. Approximately 75% of Cluster 4 overlapped with the predominantly peripheral clinical subtype, and Cluster 3 included the majority of patients in the mixed category. However, patients in Cluster 3 were in some instances subjectively classified as either predominantly central or peripheral (Fig. 1C). For reporting in the following section, Clusters 1, 2, 3, and 4 are referred to as minimally atrophic, predominantly central, mixed, and predominantly peripheral, respectively. Notably, Clusters 1 and 2 both derived from the clinically defined predominantly central group, whereas Clusters 3 and 4 overlapped with the mixed and predominantly peripheral categories. Representative cases from each cluster are illustrated in Figure 2.

### Comparisons Among Groups

Comparisons among groups are summarized in Table 2. Age, male-to-female ratio, and refraction were similar among the clusters (*P* = 0.7, *P* = 0.7, and *P* = 0.5, respectively), but disease severity varied greatly. Mild myopia was the most common refractive error, with the predominantly peripheral cluster exhibiting the highest median refractive error of –2.00 D. However, this group also showed the greatest variability (IQR, –5.00 to 0.00), likely contributing to the lack of statistical significance (*P* = 0.7363).

**Table 2.** Cluster Comparisons

Variable	Cluster 1 (9 Patients)	Cluster 2 (9 Patients)	Cluster 3 (12 Patients)	Cluster 4 (15 Patients)	Overall *P*
Clustering variables					
Macular atrophy area (mm^2^), median (IQR)	8.6 (5.2 to 12.2)	35.1 (31.1 to 41.6)	15.0 (14.8 to 18.8)	6.2 (3.2 to 8.4)	—
Angular extension (°), median (IQR)	0 (0 to 0)	9.5 (0 to 20.5)	84.2 (33.2 to 107.0)	79.5 (50.8 to 110.0)	—
Outcome variables					
Female, *n* (%)	5 (55.6)	5 (55.6)	9 (75.0)	7 (46.7)	0.521^a^
Arterial hypertension, *n* (%)	6 (66.7)	3 (33.3)	6 (60)	4 (30.8)	0.2775^a^
Glaucoma	1 (11.1)	1 (11.1)	2 (20)	1 (7.7)	0.9157^a^
Age (y), mean ± SD	65.5 ± 7.2	65.6 ± 5.9	63.1 ± 6.1	62.2 ± 12.3	0.736^b^
BCVA (logMAR), median (IQR)	0.33 (0.23 to 0.43)	0.7 (0.45 to 1.3)	0.22 (0.15 to 0.38)	0.15 (0.1 to 0.6)	0.123^c^
Spheric equivalent (D), median (IQR)	−1.25 (−1.75 to −1.00)	−0.75 (−2.25 to −0.25)	−0.5 (−2.50 to 0.00)	−2 (−5.25 to 0.00)	0.7363^c^
Foveal atrophy, *n* (%)	3 (33)	7 (77.8)	2 (16.7)	3 (20)	**0.019^a^**
Macular fibrosis, *n* (%)	1 (11.1)	6 (66.7)	2 (16.7)	1 (6.7)	**0.007^a^**
MNV development, *n* (%)	0 (0)	2 (22.2)	1 (8.3)	0 (0)	0.134^a^
Pavingstone-like degeneration, *n* (%)	0 (0)	6 (66.7)	12 (100)	15 (100)	**5.876e^−^^9^** ^a^
Distance from optic disc (mm), median (IQR)	25 (25 to 25)	21.2 (20.4 to 25)	17.8 (14.8 to 19.7)	16.0 (13.6 to 13)	**1.31e^–^^5^** ^c^
FT (µm), mean ± SD	240 ± 43.0	175 ± 58.1	199 ± 48.4	241 ± 58.4	**0.015** ^b^
SCT (µm), mean ± SD	148 ± 37.6	94.6 ± 36.7	142 ± 62.2	141 ± 45.4	0.07^b^

Summary of clusters characteristics: (1) minimally atrophic EMAP, relatively small macular atrophy area without peripheral pavingstone-like degeneration; (2) predominantly central, large macular atrophy area with no to limited peripheral atrophy; (3) mixed, intermediate macular atrophy with considerable peripheral atrophy extension; and (4) predominantly peripheral, relatively small macular atrophy with considerable peripheral atrophy extension.

Statistically significant *P* values are in bold. Pairwise *P* values adjusted with the Benjamini–Hochberg correction for tests yielding an overall *P* < 0.05 are reported separately in Supplementary Table S1.

a P value for Fisher's exact test.

b *P* value for Kruskal–Wallis.

c *P* value for ANOVA.

The predominantly central cluster showed the largest atrophy compared to mixed, predominantly peripheral, and minimally atrophic subtypes (Table 2). Similarly, this cluster exhibited the worse BCVA (logMAR) of 0.7 (IQR, 0.45–1.3), approximately corresponding to 20/100 Snellen, followed by minimally atrophic EMAP (median, 0.33; IQR, 0.23–0.43); however, patients in the mixed and predominantly peripheral groups had a relatively preserved BCVA. Differences in the clustering variables were further validated, being found statistically significant with linear mixed models by using eye-level data (Table 3). Predominantly central EMAP cases also had the highest prevalence of foveal atrophy (77.8%; *P* = 0.019), macular fibrosis (66.7%; *P* = 0.007), and MNV development (8.3%; *P* = 0.134). However, bilateral MNV in two predominantly central cases likely led to an underestimation of its prevalence in this group.

**Table 3.** Linear Mixed Model of BCVA and Macular Atrophy Area by Cluster

	Dependent Variable: BCVA (logMAR)			Dependent Variable: Macular Atrophy Area (mm^2^)		
	Estimate	95% CI	*P*	Estimate	95% CI	*P*
Cluster 2, predominantly central (reference)	—	—	—	—	—	—
Cluster 1, minimally atrophic	−0.586	(−1.028, –0.143)	**0.013**	−27.7	(−32.4, −23.0)	**1.19e** **^−^** **^14^**
Cluster 3, mixed	−0.435	(−0.867, −0.004)	0.055	−19.3	(−23.7, −14.9)	**7.43e** **^−^** **^11^**
Cluster 4, predominantly peripheral	−0.59	(−0.998, 0.182)	**0.007**	−30.4	(−34.5, −26.2)	**<2e** **^−^** **^16^**

CI, confidence interval.

Number of eyes (patients) = 85 (45). Statistically significant *P* values are in bold.

By definition, pavingstone-like degeneration was present in all patients from the mixed and predominantly peripheral clusters and in six predominantly central cases (66.7%), but was absent in minimally atrophic disease. Consequently, both its angular extension and distance from the optic disc differed among clusters, with greater involvement in the mixed and predominantly peripheral clusters, both showing a median angular extension of roughly 3 clock-hours, compared to less than 10° in predominantly central cases. However, predominantly peripheral EMAP cases exhibited comparable peripheral atrophy angular and centripetal extension to mixed cases but had smaller macular atrophy.

Consistent with previous findings, FT differed significantly among clusters (*P* = 0.015). Specifically, predominantly peripheral and minimally atrophic EMAP had similar mean FT values of 241 ± 58.4 µm and 240 ± 43.0 µm, respectively, which were higher than those observed in both mixed (199 ± 48.4 µm) and predominantly central (175 ± 58.1 µm) cases, although pairwise comparisons did not reach statistical significance after applying the Benjamini–Hochberg correction. Finally, the mean SCT of 94.6 ± 36.7 was considerably lower in predominantly central EMAP compared to the other three clusters, although this finding barely missed statistical significance (*P* = 0.07).

## Discussion

EMAP is a bilateral, symmetric retinopathy characterized by rapidly expanding macular atrophy and generalized photoreceptor dysfunction, affecting working age individuals.^2^ Multilobular macular atrophy with MDAF signal developing over diffuse sub-RPE deposits, widespread subretinal drusenoid deposits, and pseudodrusen-like lesions at the posterior pole and midperiphery are constant features of the disease. In contrast, the presence and extent of peripheral atrophy vary considerably, with direct consequences on the patients’ peripheral visual field and quality of life.^3^ Moreover, clinical manifestations often evolve over time, with some cases progressing toward a confluent pattern with panretinal involvement.^8^ Although the rapid expansion of macular atrophy has been well documented, the factors underlying the variability in peripheral involvement remain unknown, and a comprehensive understanding of the long-term progression of EMAP is still lacking.^2^^–^^7^ The identification of different EMAP subtypes may improve our understanding of the disease.

The present investigation relied on an initial subjective classification into three subtypes, based on the extent of the peripheral involvement relative to the size of the central lesion. Subsequently, hierarchical clustering demonstrated a good correspondence to the clinical judgment, further refining the predominantly central subtype into two distinct clusters—namely, Cluster 1 and Cluster 2. Drawing from their characteristics, the resulting four clusters were renamed as minimally atrophic, predominantly central, mixed, and predominantly peripheral.

The minimally atrophic cluster exhibited isolated macular atrophy with limited extension and could represent the onset of the disease whose evolutive trajectory must be defined over the follow-up. However, it is worth noting that mean age was similar across the four clusters, and EMAP patients with a later onset will also fall in this group. Minimally atrophic EMAP also may be more prone to misdiagnosis as age-related macular degeneration.^6^^,^^7^ However, clinical features such as history of night blindness and photophobia, an area of relatively spared retina temporal to the macula, rod and cone dysfunction on full-field electroretinography, and the absence of drusen can assist in differentiating the two conditions.^2^

Compared to the other clusters, predominantly central EMAP was characterized by reduced foveal sparing, lower foveal and choroidal thickness, and higher rates of MNV and subretinal fibrosis. This cluster also exhibited the poorest visual function, assessed only by BCVA, which would benefit from integration with more objective exams such as microperimetry. Peripheral atrophy was occasionally observed on UWF-CFPs taken in primary gaze in this cluster, although it was less extensive and localized farther from the optic disc than predominantly peripheral EMAP. The predominantly peripheral cluster exhibited opposite clinical features, with frequent foveal sparing and maintained visual acuity despite extensive peripheral atrophy. Mixed cases, in contrast, shared a similar degree of peripheral involvement with this group but exhibited greater macular involvement. Specifically, this cluster had a slightly larger atrophy area despite similar BCVA, SCT and prevalence of foveal sparing. The mixed cluster also mismatched the most with the subjective classification, highlighting the need for longitudinal studies to determine whether mixed cases represent a transitional stage, the severe end of predominantly peripheral types, or a distinct disease trajectory.

To this date, EMAP etiopathogenesis remains elusive, with hypotheses including prolonged exposition to toxic environmental factors,^10^^,^^11^ a pro-inflammatory or autoimmune milieu,^10^^,^^12^^–^^14^ impaired choroidal perfusion,^7^^,^^8^^,^^15^^,^^16^ and monogenic inheritance. We suppose that multiple mechanisms contribute to the development of EMAP and that the interplay among these unknown factors may drive the emergence of distinct clinical subtypes. For example, although the degree of myopia was similar among the groups, the predominantly peripheral cluster covered a wide range of refractive errors, with one quarter of the patients having a refractive error higher than –5.25 D. Thus, it is possible that anatomical factors such as axial length play a role in determining the formation of peripheral pavingstone-like degeneration. Alternatively, inadequate choroidal blood supply anterior to the equator of the eye could participate in the expansion of peripheral atrophy.^17^

This study has several limitations, including the limited number of patients, the lack of longitudinal follow-up and information on disease duration, and technical constraints inherent to UWF-CFP. In particular, the assessment of peripheral involvement was approximated based on the closest edge of the peripheral atrophy relative to the optic disc and angular extension taken on fundus photographs acquired in the primary gaze position. Additionally, using a single grader to measure atrophy areas may introduce bias although macular atrophy measurements generally show high intergrader agreement when confined to the posterior pole.^2^ Moreover, a longitudinal study is necessary to understand the prognostic relevance and clinical course of the disease subtypes identified in the present study.

In essence, we identified four subgroups through a clustering-based approach, with the predominantly central and predominantly peripheral clusters standing out as the most defined, and the mixed and minimally atrophic subgroups requiring further refinement through longitudinal studies. If confirmed through longitudinal follow-up, these findings suggest that, although macular atrophy in EMAP ensues and enlarges through a basal laminar deposit-driven process,^18^ different pathogenic factors may give rise to distinct disease subtypes, carrying implications for patient prognosis and the development of future therapeutic strategies.

## Supplementary Material

Supplement 1

Supplement 2
