# Supplementary material for: Clinical and Clustering-Based Subtyping of Extensive Macular Atrophy With Pseudodrusen-Like Appearance (EMAP)
Source: Transl Vis Sci Technol. 2025 Dec 24;14(12):26. doi: 10.1167/tvst.14.12.26 (PMC12743488; doi:10.1167/tvst.14.12.26)
Supplement: Supplement 1 [file tvst-14-12-26_s001.docx]

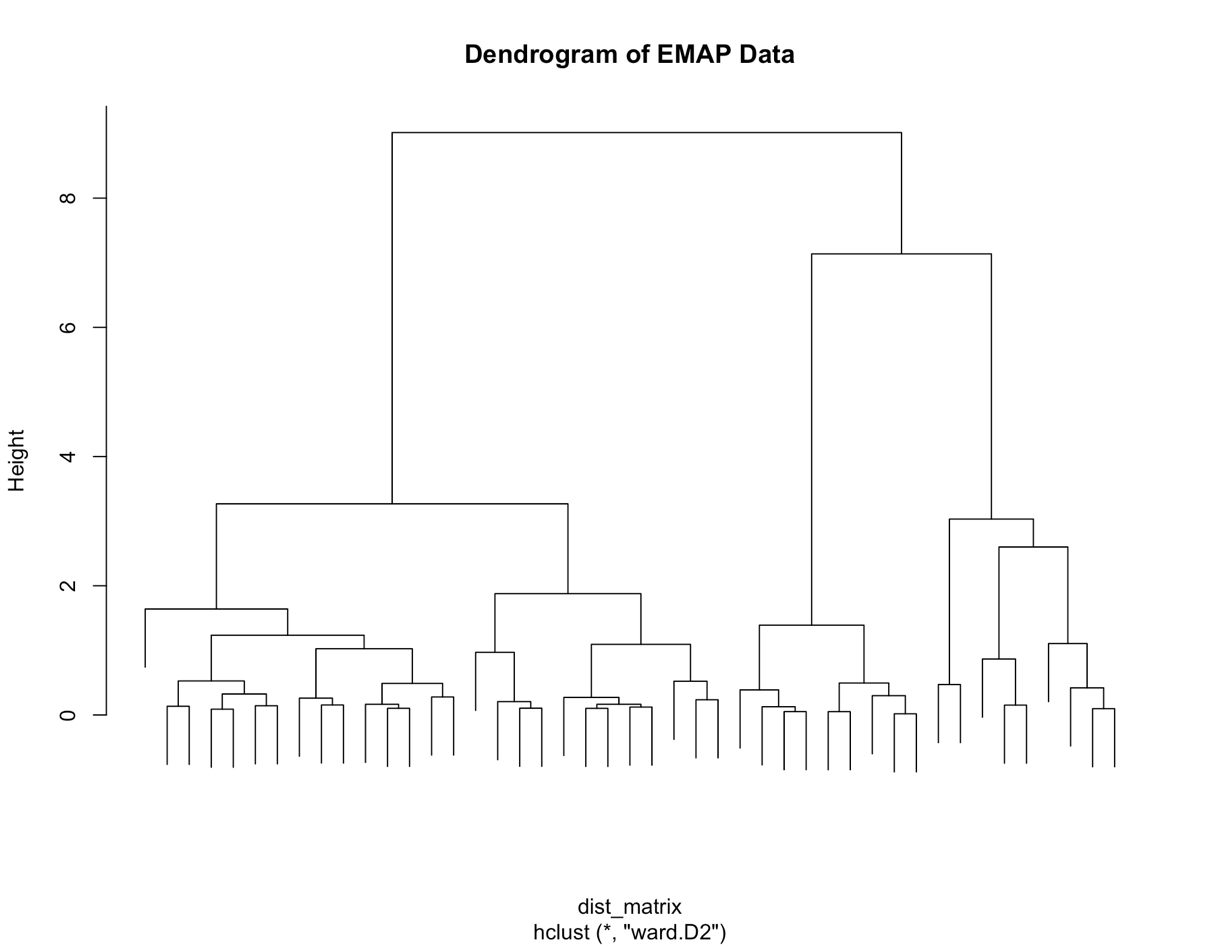


**Supplemental Figure 1.** Dendrogram of EMAP cases based on hierarchical clustering using Ward’s method and a Euclidean distance matrix.


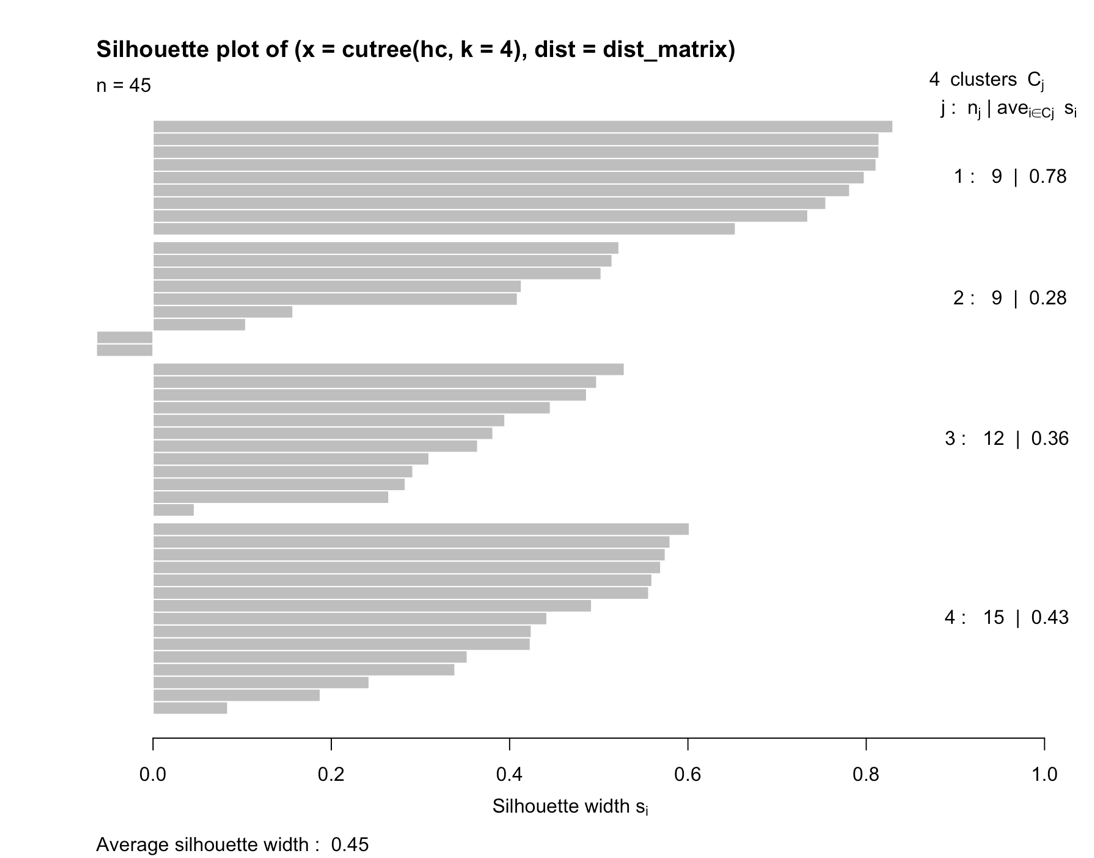


**Supplemental Figure 2.** Silhouette plot showing the cohesion and separation of the four clusters identified, with an average silhouette width of 0.45.
