# Supplementary material for: Clinical and Clustering-Based Subtyping of Extensive Macular Atrophy With Pseudodrusen-Like Appearance (EMAP)
Source: Transl Vis Sci Technol. 2025 Dec 24;14(12):26. doi: 10.1167/tvst.14.12.26 (PMC12743488; doi:10.1167/tvst.14.12.26)
Supplement: Supplement 2 [file tvst-14-12-26_s002.docx]

| **Supplementary Table S1. Pairwise comparisons by cluster** | | | | | | | | |
| --- | --- | --- | --- | --- | --- | --- | --- | --- |
| **Outcome variable** | **Statistical test** |  | **Pairwise cluster comparisons** | | | | | |
|  |  | **Overall p** | *1 vs 2* | *1 vs 3* | *1 vs 4* | *2 vs 3* | *2 vs 4* | *3 vs 4* |
| Distance from optic disc (mm) | Kruskal-Wallis | **1.31e^-5^** | **0.011** | **0.0002** | **0.0002** | **0.05** | **0.01** | 0.15 |
| Foveal thickness (µm) | ANOVA | **0.015** | 0.18 | 0.44 | 0.99 | 0.90 | 0.16 | 0.37 |
| Statistically significant p values in bold. Pairwise p values were adjusted with the Benjamini- Hochberg correction and were only reported if overall test’s p < 0.05 | | | | | | | | |
